# Case for diagnosis. Single-digit clubbing^☆^^☆☆^

**DOI:** 10.1016/j.abd.2020.01.006

**Published:** 2020-05-11

**Authors:** Larissa Crestani, Isaura Azevedo Fasciani, Priscila Kakizaki, Neusa Yuriko Sakai Valente

**Affiliations:** Department of Dermatology, Hospital do Servidor Público Estadual de São Paulo, São Paulo, SP, Brazil

**Keywords:** Connective tissue, Fingers, Solitary fibrous tumors

## Abstract

A 58-year-old female patient presented with a single-digit clubbing on the second finger of her right hand two years previously. After investigation with imaging and incisional biopsy, superficial acral fibromyxoma was diagnosed. A brief review on single-digit clubbing and its causes is presented, focusing on superficial acral fibromyxoma.

## Case report

**Figure 1:**
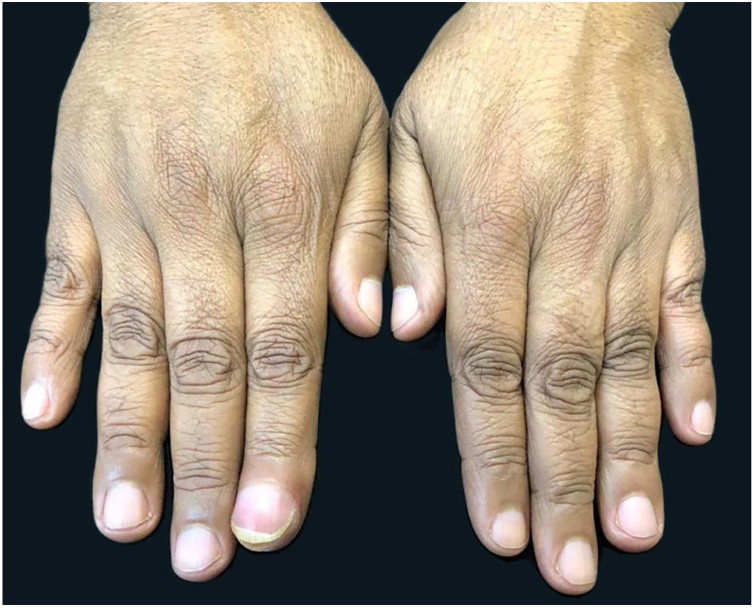
Increased volume of the second finger in the right hand.

**Figure 2:**
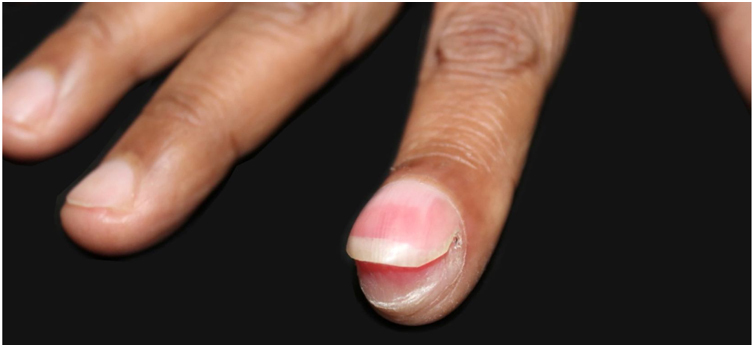
Hypertrophy of the distal phalanx associated with increased nail bed convexity.

**Figure 3:**
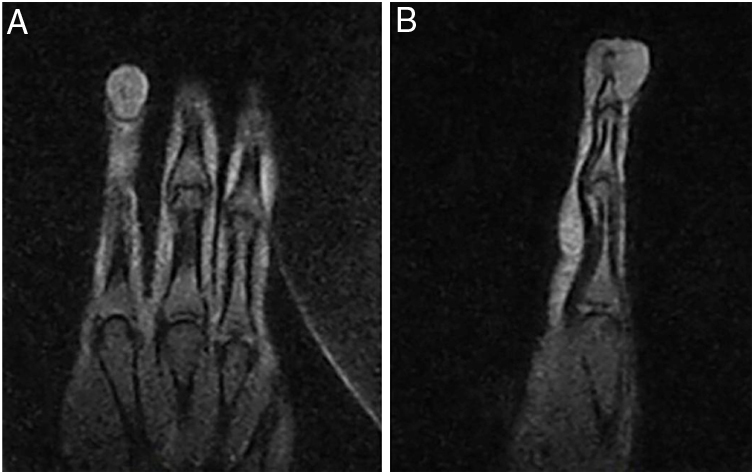
Nodular formation on the dorsal surface of the distal segment of the finger, located superficially to the phalanx, causing adjacent bone remodeling and bulging the skin surface.

**Figure 4:**
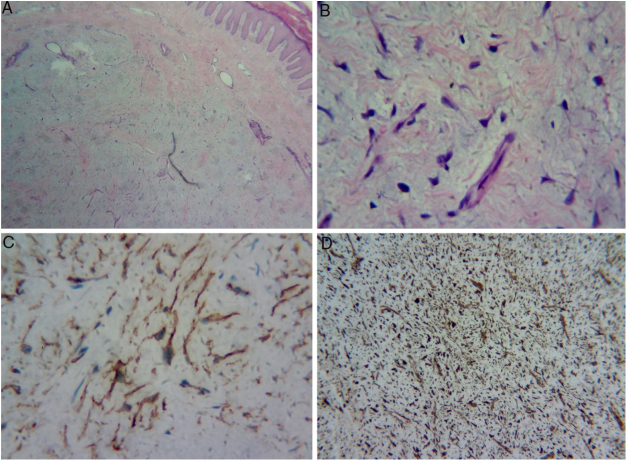
(A) Dermal proliferation of spindle and stellate cells without atypia, immersed in a myxoid stroma with moderate proliferation of small vessels. (B) Greater magnification showing the stellate cells. (C) Positive immunohistochemistry exam for CD34. (D) Positive immunohistochemistry exam for vimentin in the cytoplasm of all cells.

A 58-year-old hypertensive woman reported a progressive increase in volume in the distal phalanx of the second right finger for two years, asymptomatically. On examination, she presented hypertrophy of the distal phalanx associated with increased nail bed convexity, suggesting single-digit clubbing (Figure 1, Figure 2) and confirmed by the profile angle and phalangeal depth ratio.^1^ Nuclear magnetic resonance showed a nodular formation on the dorsal aspect of the distal segment of the finger, located superficially to the phalanx, causing adjacent bone remodeling, measuring 1.8 × 1.3 × 1.0 cm and causing bulging of the skin surface (Fig. 3). Lesion biopsy was performed (Fig. 4).

**What is your diagnosis?**

A. Enchondroma
B. Osteoid osteoma
C. Myxoid cyst
D. Superficial acral fibromyxoma

## Discussion

Histopathology assessment showed dermal proliferation of spindle and stellate cells without atypia, within a myxoid stroma with moderate proliferation of small vessels, suggestive of neurofibroma or superficial acral fibromyxoma (SAFM). Immunohistochemistry assessment was requested to distinguish between the two tumors; cells positive for vimentin, CD34, and KI-67 (1%), and negative for S-100 (Fig. 4) were observed. Negativity for S-100 protein and positivity for CD34 favored the diagnosis of SAFM.

Digital clubbing is characterized by a focal increase in the terminal segments of the fingers due to the proliferation of connective tissue between the nail matrix and the distal phalanx. For confirmation, it is recommended to calculate the profile angle, which should be greater than 180°, and the ratio of the depth of the distal phalanx to the interphalangeal joint, which should be greater than 1.^1^, ^2^

Single-digit clubbing is a rare condition, usually caused by an expansive process in the distal phalanx. Enchondromas, osteoid osteoma, myxoid cyst, and myxochondromas have been described as the cause of this condition.^2^ To the best of the authors’ knowledge, the literature presents only one case of single-digit clubbing due to SAFM.^3^

SAFM is a rare and benign soft tissue tumor, usually with slow and painless growth. It usually affects the periungual and subungual regions of the fingers and toes. Its radiographic findings are nonspecific.^4^, ^5^

Histologically, there is dermal or subcutaneous proliferation of fusiform and stellate cells within a myxoid, mixoid-collagenous, or collagenous matrix, with prominent microvasculature and mast cell infiltrate. Mild nuclear atypias and mitoses may be observed. Immunohistochemistry assessments are positive for CD34, CD99, vimentin, and CD10, and negative for S-100.^4^, ^5^, ^6^, ^7^, ^8^

Differential diagnoses include myxoid tumors and those that affect the distal part of the limbs, such as dermatofibrosarcoma protuberans, myxoid neurofibroma, fibrous histiocytoma, acquired digital fibrokeratoma, acral myxoinflammatory fibroblastic sarcoma, sclerosing perineurioma, superficial angiomyxoma, and low-grade fibromyxoid sarcoma, in addition to the previously mentioned causes of single-digit clubbing.^4^, ^7^, ^8^, ^9^

In acquired digital fibrokeratoma, CD34 may be positive, but it differs histopathologically, as it presents pronounced hyperkeratosis and acanthosis, low cellularity, and thick collagen bundles parallel to the long axis of the lesion. Low-grade fibromyxoid sarcoma is negative for CD34, unlike SAFM, which is positive.^10^

Treatment consists of complete excision of the lesion.^4^, ^5^, ^7^ The patient opted for clinical follow-up due to the benign nature of the lesion.

## Financial support

None declared.

## Authors’ contributions

Larissa Crestani: Approval of the final version of the manuscript; elaboration and writing of the manuscript; intellectual participation in propaedeutic and/or therapeutic conduct of studied cases; critical review of the literature; critical review of the manuscript.

Isaura Azevedo Fasciani: Critical review of the literature; critical review of the manuscript.

Priscila Kakizaki: Approval of the final version of the manuscript; intellectual participation in propaedeutic and/or therapeutic conduct of studied cases; critical review of the literature; critical review of the manuscript.

Neusa Yuriko Sakai Valente: Approval of the final version of the manuscript; intellectual participation in propaedeutic and/or therapeutic conduct of studied cases; critical review of the literature; critical review of the manuscript.

## Conflicts of interest

None declared.
